# Supplementary material for: DMRforPairs: identifying Differentially Methylated Regions between unique samples using array based methylation profiles
Source: BMC Bioinformatics. 2014 May 15;15:141. doi: 10.1186/1471-2105-15-141 (PMC4046028; doi:10.1186/1471-2105-15-141)
Supplement: Additional file 2 — DMRforPairs output for the comparison of A431-MCF7 and NA17018-NA17105. Please start from the HTML files in each folder. Available via the BMC Bioinformatics website. [file 1471-2105-15-141-S2.zip › 1394847754114233_MOESM2_ESM/A431_MCF7/figures/13248.pdf]

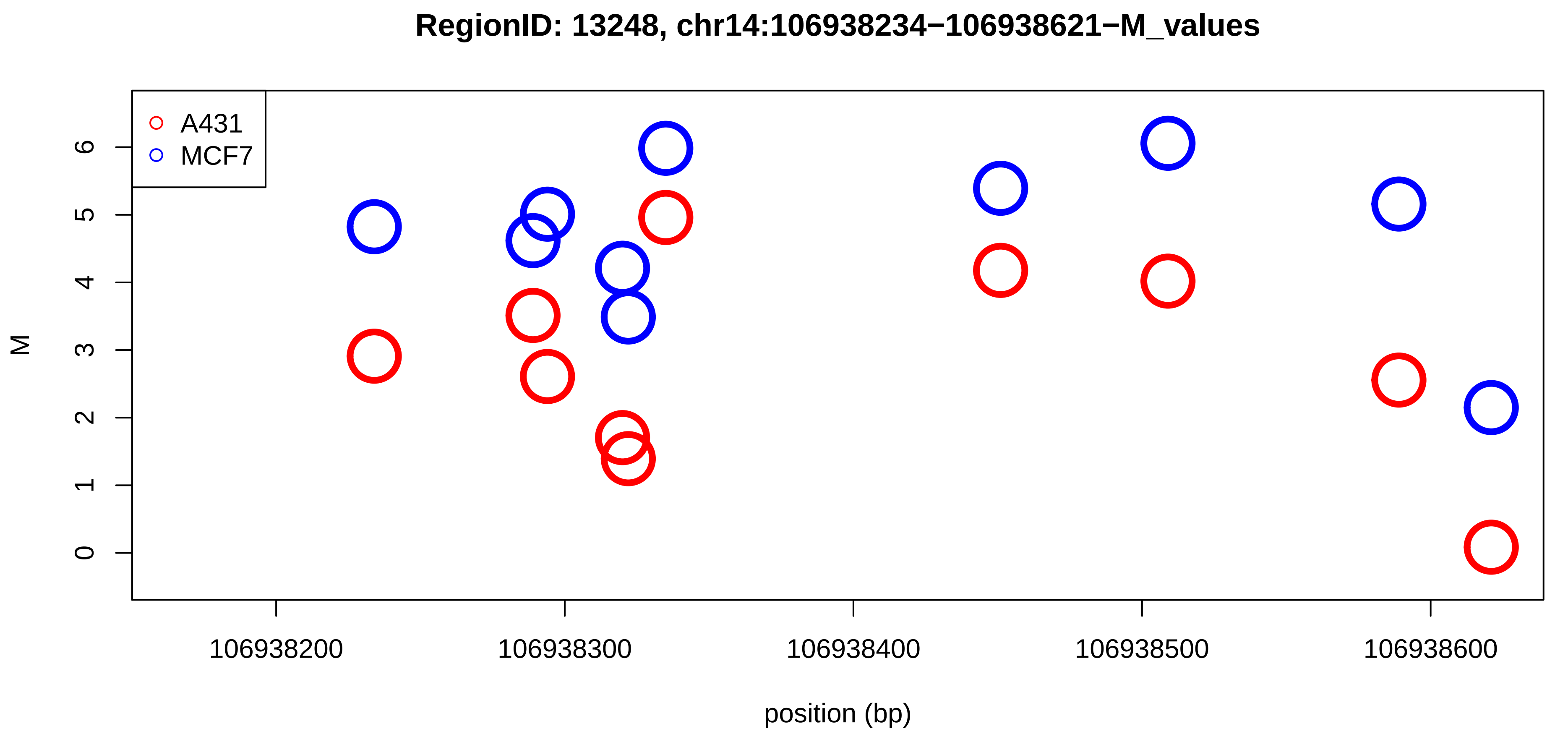

RegionID: 13248, chr14:106938234−106938621−M_values
A431
MCF7
M
0
1
2
3
4
5
6
106938200
106938300
106938400
106938500
106938600
position (bp)

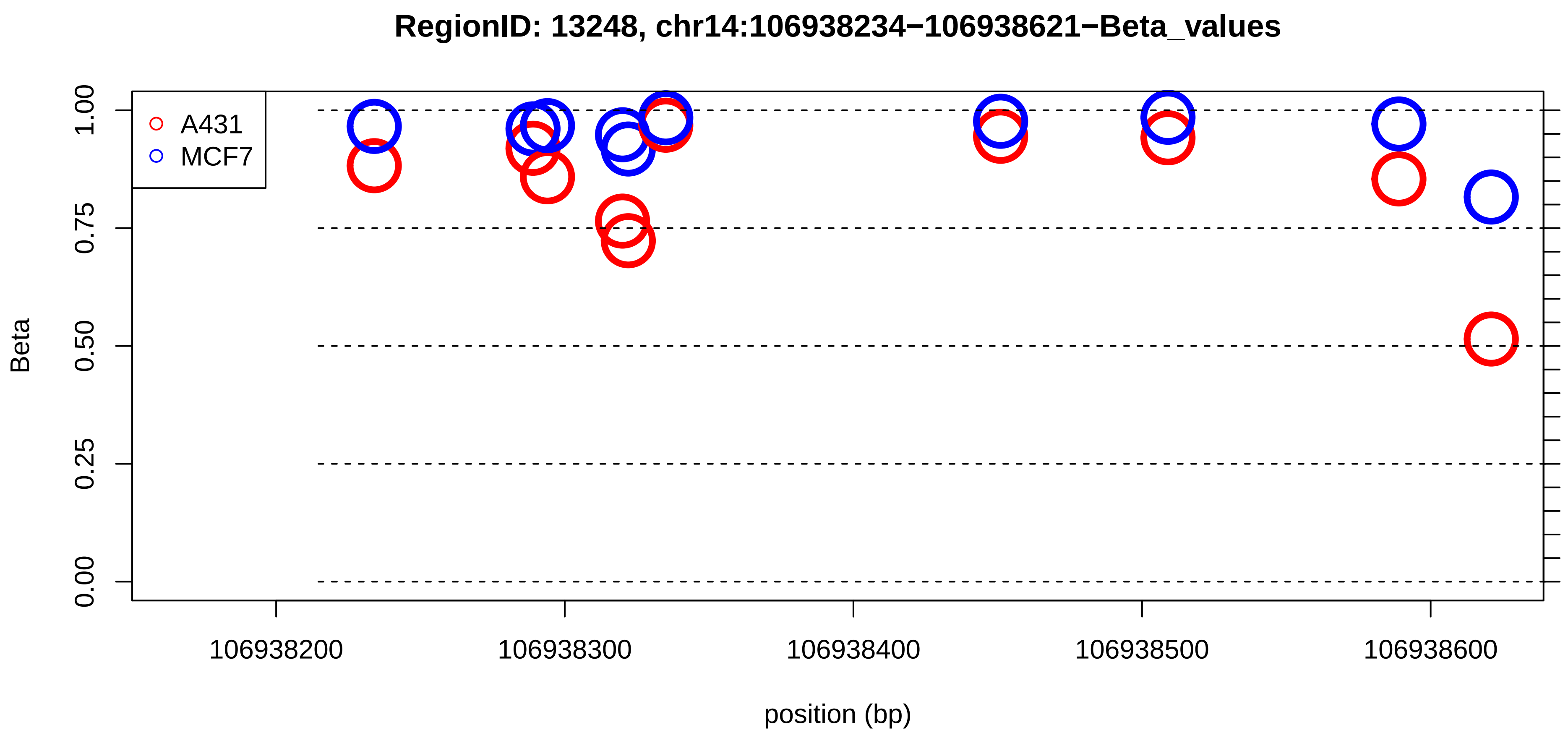

RegionID: 13248, chr14:106938234−106938621−Beta_values
A431
MCF7
Beta
0.00
0.25
0.50
0.75
1.00
106938200
106938300
106938400
106938500
106938600
position (bp)

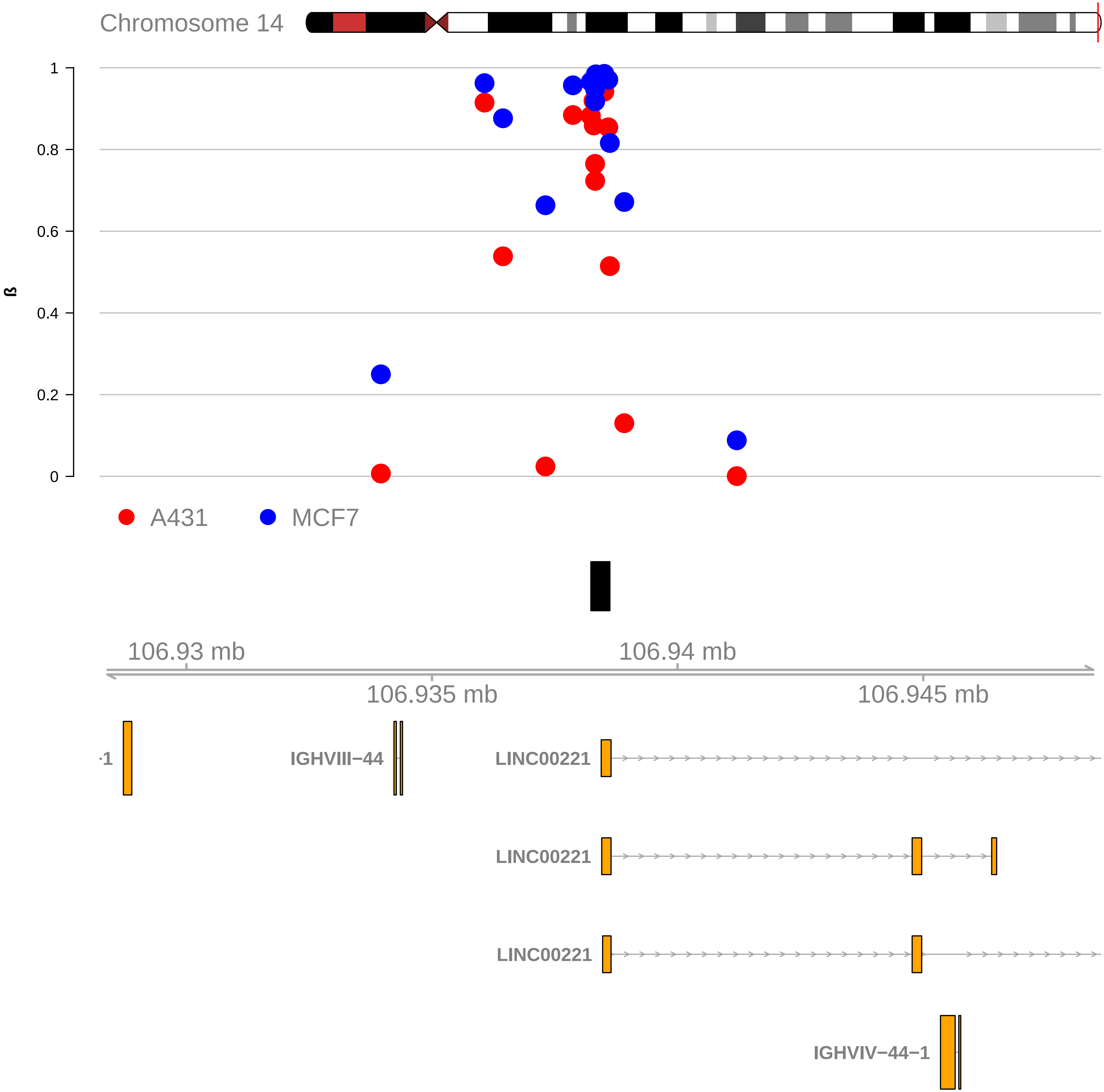

Chromosome 14
1
0.8
0.6
0.4
0.2
0
ß
A431
MCF7
106.93 mb
106.94 mb
106.935 mb
106.945 mb
-1
IGHVIII−44
LINC00221
LINC00221
LINC00221
IGHVIV−44−1
